# Supplementary material for: Effects of Policy for Controlling Agricultural Non-Point Source Pollution in China: From a Perspective of Regional and Policy Measures Differences
Source: Int J Environ Res Public Health. 2023 Feb 20;20(4):3741. doi: 10.3390/ijerph20043741 (PMC9967139; doi:10.3390/ijerph20043741)

Appendix S1: The loading coefficients for all sources

Table S1: The loading coefficients of livestock and poultry breeding

Region	Livestock and Poultry	Pollutant index	Loading coefficients	Region	Type	Pollutant index	Loading coefficients
North China Region	Pig	COD	109.02	South Central Region	Pig	COD	84.66
		TN	16.92			TN	11.65
		TP	1.70			TP	1.32
	Cattle	COD	924.86		Cattle	COD	1178.78
		TN	44.72			TN	70.71
		TP	7.32			TP	8.13
	Chicken	COD	5.11		Chicken	COD	2.78
		TN	30.57			TN	0.16
		TP	0.06			TP	0.04
Northeast China Region	Pig	COD	121.56	Southwest China Region	Pig	COD	88.38
		TN	17.47			TN	13.04
		TP	2.03			TP	1.01
	Cattle	COD	1201.29		Cattle	COD	595.69
		TN	67.15			TN	35.47
		TP	9.76			TP	4.33
	Chicken	COD	6.78		Chicken	COD	2.51
		TN	0.37			TN	0.10
		TP	0.22			TP	0.03
East China Region	Pig	COD	87.31	Northwest China Region	Pig	COD	90.39
		TN	4.89			TN	13.30
		TP	1.02			TP	4.47
	Cattle	COD	944.63		Cattle	COD	816.75
		TN	55.04			TN	55.61
		TP	7.01			TP	3.90
	Chicken	COD	8.16		Chicken	COD	4.98
		TN	0.28			TN	0.25
		TP	0.11			TP	0.07

Table S2: The loading coefficients of freshwater aquaculture

Region	TN	TP	COD
Beijing	17.32577	3.770467	47.40677
Tianjin	22.869	4.311128	29.86797
Hebei	21.46905	4.208316	29.59745
Shanxi	24.12041	4.795588	30.91124
Inner Mongolia	22.1592	4.3391	47.51065
Heilongjiang	9.330632	1.506105	51.47626
Jilin	14.786	2.889111	35.91274
Liaoning	18.20425	3.234886	47.06705
Shandong	18.16071	3.700346	48.79479

Shanghai	7.020194	1.294484	49.15971
Zhejiang	23.18479	4.48744	58.67741
Henan	19.03191	3.320907	50.54672
Anhui	21.48954	4.072463	54.36843
Hubei	19.40327	3.594358	55.6647
Hunan	17.24303	3.176033	53.49295
Jiangxi	19.17847	3.3726	58.56709
Guangdong	12.85422	2.511782	43.56718
Guangxi	16.94109	3.129279	37.53458
Hainan	11.97184	2.14444	47.33664
Chongqing	14.28302	2.392163	41.28195
Sichuan	19.15205	3.640309	54.48936
Guizhou	20.45242	4.197	41.23777
Yunnan	13.42151	2.522027	49.63176
Shanxi	17.26937	3.454789	34.40142
Ningxia	6.346778	1.109778	47.38044
Gansu	15.41353	3.291733	27.79327
Qinghai	8.648	1.724	58.119
Xinjiang	11.42509	2.91213	73.36935
Jiangsu	20.67275	4.093893	53.7361
Fujian	18.0195	3.499611	51.1223
Tibet	13.16166	2.699617	58.90237

Note: The value of Jiangsu is the mean value of Zhejiang and Shandong, the value of Fujian is the mean value of Zhejiang and Guangdong, and the value of Xizang is the mean value of Xinjiang, Sichuan, Qinghai, and Yunnan

Table S3: The loading coefficients of aquaculture

Region	TN	TP	COD
Tianjin	14.83235	3.402059	52.12741
Hebei	3.671389	0.968	43.58794
Liaoning	12.67976	1.653476	42.84348
Shandong	17.53933	2.722194	41.136
Shanghai	5.972667	0.353667	8.989667
Zhejiang	19.8597	3.12345	39.14138
Guangdong	15.6859	2.687	44.13631
Guangxi	30.15156	4.534813	57.39775
Hainan	24.932	4.1945	52.39333
Jiangsu	18.69952	2.922822	40.13869
Fujian	17.7728	2.905225	41.63884

Note: The value of Jiangsu is the mean of Zhejiang and Shandong, and the value of Fujian is the mean of Zhejiang and Guangdong

Table S4: The loading coefficients of fertilizer

Region	TN	TP
Northern Plateau Mountain region	0.293%	0.215%
Northeast China Subhumid Plain region	0.422%	0.096%
Huang-huai-hai subhumid plain area	1.173%	0.199%
Southern China Mountainous and Hilly region	0.868%	0.497%
Southern humid plain area	1.536%	0.410%
Northwest China Arid and Semi-Arid Plain region	0.511%	0.108%

Table S5: The loading coefficients of crop residue

Region	COD	TN	TP
Northern Plateau Mountain region	11.7500%	10.1500%	9.8760%
Northeast China Subhumid Plain region	8.5533%	6.9317%	6.6173%
Huang-huai-hai subhumid plain area	12.7975%	10.7825%	8.6495%
Southern China Mountainous and Hilly region	9.6989%	7.7978%	6.9351%
Southern humid plain area	9.7000%	7.8667%	7.6127%
Northwest China Arid and Semi-Arid Plain region	6.1050%	5.2600%	6.1900%

Table S6: The loading coefficients of rural domestic sewage pollution

Pollutant	The loading coefficients
TN	0.89
TP	0.20
COD	7.82

Appendix S2: Measurement of agricultural non-point source pollution by entropy method

The paper determined the weights of TN, TP, and COD discharges on the foundation of TN, TP, and COD emissions in the agricultural field in 31 provinces (municipalities and autonomous regions) from 2010 to 2019. Moreover, the paper integrated the three pollution indicators into one index "agricultural non-point source pollution level" by using the entropy method. The specific methods are as follows:

1. Index selection: Suppose there are r years, n provinces (municipalities, autonomous regions), and m indexes. x_{itj} is the Jth index of province i in the t year.

2. Indicators standardization processing: Positive indicators: $x'_{itj} = x_{itj}/x_{max}$, Negative indicators: $x'_{itj} = x_{min}/x_{itj}$,
3. Calculation indicator weights: $y_{itj} = x'_{itj} / \sum_{\theta} \sum_{i} x'_{itj}$.
4. Calculate the entropy of the Jth index: $e_j = -k \sum_t \sum_i y_{itj} * \ln(y_{itj})$, which k>0, k=ln(rn)
5. Calculate the information utility value of the j-th index: $g_j = 1 - e_j$.
6. Calculate the weight of each indicator: $w_j = g_j / \sum_j g_j$.
7. Calculate the comprehensive score of agricultural non-point source pollution level in provinces (municipalities and autonomous regions): $ER_{it} = \sum_j (w_j\, x'_{itj})$.

In theory, the TN, TP, and COD discharge as undesirable output should be negative indicators. However, the index "agricultural non-point source pollution level" constructed based on the entropy method in this study is also an unexpected index, that is, the discharge index of the three types of pollutants and the index of "agricultural non-point source pollution level" is in the same direction, so the standardization of the discharge index of the three types of pollutants should be treated in accordance with the standardization of positive indicators.

Appendix S3: The scoring criteria of policy strength calculation

Table S7: The details of the scoring criteria of seven indicators for policy strength calculation

**Policy intensity**	
Score	Evaluation criteria
4	Local laws and regulations.
3	Local government regulations.
2	Local normative documents.
1	Local working file.
**Policy objectives**	
Score	Evaluation criteria
4	Documents that address agricultural non-point source pollution or the treatment of an agricultural pollution source and sets quantifiable targets.
3	Documents for the treatment of agricultural non-point source pollution or an agricultural pollution source without quantifiable targets.
2	The objective part of the documents includes agricultural non-point source pollution or the control of a certain agricultural pollution source and puts forward quantifiable objectives.
1	The objective part of the documents includes agricultural non-point source pollution or the control of a certain agricultural pollution source but does not put forward quantifiable targets.
**Policy monitoring**	
Score	Evaluation criteria
3	Mention that inspections should be carried out and specify specific inspection methods.

2	The reference to inspection was made without specifying the specific method of inspection.
1	There are no environmental regulatory inspections.
**Policy measures**	
Score	Evaluation criteria（**Administrative regulation**）
5	Explicitly prohibit the use of an action or a product. For example, it is forbidden to discharge irrigation water directly into water bodies and to apply organic fertilizers without harmless treatment into farmland.
4	Use quantifiable regulatory instruments. For example, limit breeding density and standardize the use of agricultural inputs.
3	Use unquantifiable regulatory means but give a clear governance scheme. Such as formulating plans, establishing systems, and promoting the construction of the pilot (demonstration zone).
2	Only from the macro level mentioned relevant content, the proposed plan and measures are not clear. For example, units and individuals engaged in agricultural production may not pollute the agricultural environment.
1	Command control measures are not listed.
Score	Evaluation criteria（**Economic incentive**）
5	Specify specific economic incentives (taxes, subsidies, fines, etc.) and specify the amount or proportion of funds.
4	Specific economic incentives (taxes, subsidies, fines, etc.) are specified, but the amount or proportion of funds is not specified.
3	When it comes to financial support, there are clear rules, but the funds can be used more widely. For example, to allocate capital construction funds for the control of pollution from non-point agricultural sources, the appropriation for the protection of the agricultural ecological environment shall be included in the fiscal budget.
2	Talk of economic support, but no clear rules or concrete measures. For example, guiding social capital investment.
1	No economic incentives are listed.
Score	Evaluation criteria（**Technical support**）
5	Referring to a specific scientific technology, such as soil testing and fertilizer formulation.
4	Mention science and technology, but the technology is not clear. Such as straw raw material utilization.
3	Measures to promote the development of agricultural environmental protection technology were put forward. For example, the establishment of a technical information-sharing platform, promotes "industry-university-research" cooperation.
2	It only mentions supporting scientific and technological development. For example, the state encourages and supports the development and application of relevant technologies.
1	No technical support measures are listed.
Score	Evaluation criteria（**Educational**）
5	Mention clear and direct communication and education methods and content. For example, training farmers on the skills of harmless treatment of livestock and poultry waste.
4	Mention explicit and indirect methods and contents of education. Such as the use of radio and television propaganda through straw comprehensive utilization knowledge.
3	There is no specific content of propaganda and education, or there is no specific content of propaganda and education.
2	Only from the macro level mentioned to strengthen environmental awareness, there is no specific operation plan and specific publicity and education content.
1	No information or educational measures are listed.

Appendix S4: The differences in policy documents

Local judicial documents: government departments in the investigation, prosecution, trial, notarization, and other judicial organs in the handling of various cases in each link and step of the formation and use of special documents.

Administrative licensing approval documents: a written reply document in which the people's government, after examining the application submitted by the applicant, decides whether to grant or not to grant the administrative license.

Local regulations: the normative documents formulated and promulgated by the statutory local organs of state power in accordance with the statutory limits of authority and on the premise that they are not in conflict with the Constitution, laws, and administrative regulations.

Local government rules: Local people's governments may enact rules in accordance with national laws, administrative regulations, and local decrees of their provinces, autonomous regions, and municipalities.

Local normative documents: Because its content has the nature of restricting and regulating people's behavior, the name is normative documents. It refers to the legislative documents and non-legislative documents formed by the authority in the process of performing their duties, which have specific effectiveness and normative format and can be applied repeatedly. The content of these documents has the nature of constraining and regulating people's behavior.

Local working documents: they are normative documents concerning the internal work arrangement of the government and the rights and interests of citizens and enterprises.

Appendix S5: The location of the regions for the dataset in China

Figure S1: The location of the regions for the dataset.

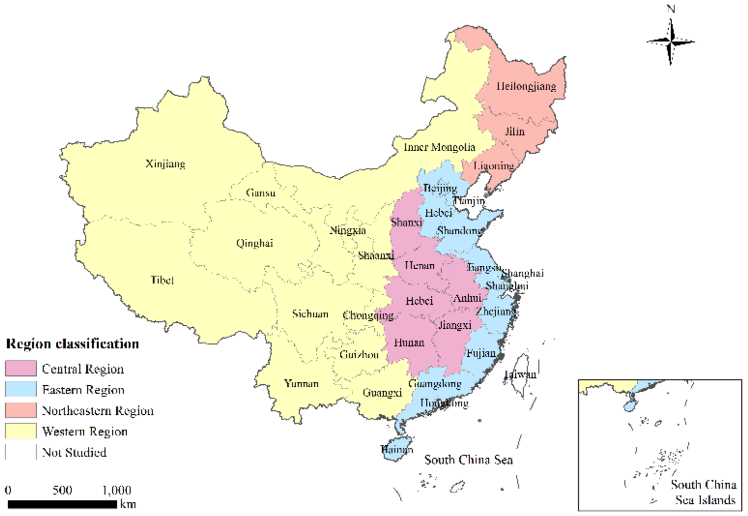

Supplement: Supplementary file 1 [file ijerph-20-03741-s001.zip › ijerph-2205008-supplementary.pdf]
